# Ni(II) Complexes with Schiff Base Ligands: Preparation, Characterization, DNA/Protein Interaction and Cytotoxicity Studies

**DOI:** 10.3390/molecules22101772

**Published:** 2017-10-24

**Authors:** Hui Yu, Wei Zhang, Qing Yu, Fu-Ping Huang, He-Dong Bian, Hong Liang

**Affiliations:** 1 Guangxi Key Laboratory of Chemistry and Engineering of Forest Products, School of Chemistry and Chemical Engineering, Guangxi University for Nationalities, Nanning 530008, China; yh9930228@163.com; 2 Key Laboratory for the Chemistry and Molecular Engineering of Medicinal Resources (Ministry of Education of China), School of Chemistry and Pharmacy, Guangxi Normal University, Guilin 541004, China; ZW574873607@163.com (W.Z.); gxqbs_qing@163.com (Q.Y.); hliang@gxnu.edu.cn (H.L.)

**Keywords:** Schiff base, calf thymus DNA, antitumor activity, Ni(II) metal complexes

## Abstract

In this study, two Ni(II) complexes, namely [Ni(HL1)_2_(OAc)_2_] (**1**) and [Ni(L2)_2_] (**2**) (where HL1 and HL2 are (*E*)-1-((1-(2-hydroxyethyl)-1*H*-pyrazol-5-ylimino)methyl)-naphthalen-2-ol) and (*E*)-ethyl-5-((2-hydroxynaphthalen-1-yl)methyleneamino)-1-methyl-1*H*-pyrazole-4-carboxylate, respectively), were synthesized and characterized by X-ray crystallography, Electrospray Ionization Mass Spectrometry (ESI-MS), elemental analysis, and IR. Their uptake in biological macromolecules and cancer cells were preliminarily investigated through electronic absorption (UV-Vis), circular dichroism (CD) and fluorescence quenching measurements. Bovine serum albumin (BSA) interaction experiments were investigated by spectroscopy which showed that the complexes and ligands could quench the intrinsic fluorescence of BSA through an obvious static quenching process. The spectroscopic studies indicated that these complexes could bind to DNA via groove, non-covalent, and electrostatic interactions. Furthermore, in vitro methyl thiazolyl tetrazolium (MTT) assays and Annexin V/PI flow cytometry experiments were performed to assess the antitumor capacity of the complexes against eight cell lines. The results show that both of the complexes possess reasonable cytotoxicities.

## 1. Introduction

Nowadays, cancer is gradually becoming the second most significant mortality factor after cardiovascular diseases, causing roughly a quarter of all deaths around the world, with a death rate that is increasing year by year. Therefore, it is urgent for medicinal chemists to develop new antitumor drugs that are highly active, with lower toxicity and specific selectivity [1]. Since 1965, cisplatin [2] and many other platinum-based drugs have been discovered and used as promising anticancer agents. Nevertheless, their well-known drawbacks, such as multifactorial resistance, generally low solubility and general toxicity, have restricted their wider use in cancer chemotherapy [3,4]. Thus, more effective and less toxic non-platinum metal-based anticancer complexes are being actively sought and developed [5,6,7].

In recent years, the coordination of Schiff bases to transition metal ions has been extensively studied in medicine and diagnostics [8]. The substituted heterocyclic moiety in combination with transition metal salts generate coordination compounds which possess enhanced physiochemical and pharmacological properties [9,10,11,12]. Given the high costs and toxicity of platinum-based drugs, researchers have paid more attention to transition metals, such as Ni. Nickel is recognized as an essential trace element because several hydrogenases and carbon monoxide dehydrogenases [13] contain nickel ions in the active site. More specifically, nickel complexes with varied biological activities have been reported in the literature, for instance, with antibacterial [14,15,16], antifungal [15] and antiproliferative/anticancer properties [17,18,19,20].

In this article, two Schiff base ligands (HL1 and HL2) have been prepared by condensation of 2-hydroxy-1-naphthaldehyde and 5-aminopyrazoles with hydroxyethyl and ethoxycarbonyl groups, respectively. The Ni(II) complexes with the two ligands have been synthesized, and characterized by X-ray diffraction. The cytotoxic properties of the ligands and their Ni(II) complexes in vitro have been determined. Differences between their biological activities were demonstrated through studying their binding with biological macromolecules and antitumor activities. We hope our work will lead to further evaluation of these compounds as chemotherapeutic agents for human tumours.

## 2. Results and Discussion

### 2.1. Spectroscopic Characterization

In the IR spectra of the compounds, the bands at 2845–3105 cm^−1^ are assigned to C–H stretching absorptions. The IR spectra of the Schiff base ligands HL1 (*E*)-1-((1-(2-hydroxyethyl)-1*H*-pyrazol-5-ylimino)methyl)-naphthalen-2-ol) and (*E*)-ethyl-5-((2-hydroxynaphthalen-1-yl)methyleneamino)-1-methyl-1*H*-pyrazole-4-carboxylate show sharp strong bands at around 1622 and 1624 cm^−1^, respectively, which are characteristic of C=N bands. No significant change was found in this band position in complex **1**. As a result of coordination, the spectral band was shifted to lower frequency in **2**, indicating that the amido nitrogen atom takes part in the coordination with the nickel ion [21]. In addition, the hydroxyl groups (–OH) of the Schiff bases show the absorption bands in the region of 3203–3430 cm^−1^. However, the corresponding strong absorption of *ν*(OH) for **1** and **2** were exhibited at around 3420 cm^−1^.

The UV-Vis absorption spectra for both HL1, HL2, **1** and **2** were recorded in solid state and aqueous solution at room temperature. The spectra of HL1 and HL2 reveal significantly intense regions of absorbance at around 208 nm, which can be attributed to π → π* transitions of the ligands. Compared with their ligands, the visible spectrum of **1** and **2** show typical bands of octahedral and square Ni(II) at 600 and 650 nm, respectively. These bands are assigned to ^3^A_2g_ → ^3^T_1g_, and ^1^B_1g_ → ^1^A_1g_ transitions, respectively. The stability of all complexes in DMSO and Tris-HCl buffer has been determined via UV-Vis (shown in Figure S1) spectroscopy. No distinct UV-Vis changes for any complexes in test were found for 48 h and just tiny changes in the absorptive intensities were observed, confirming the stability of all complexes at room temperature. Therefore, the solutions were allowed to employ further experiments.

The mass spectra of ligands HL1 and HL2 show similar fragmentation patterns and give molecular ion peaks [M]^+^ at *m*/*z* 280 and 322.9, respectively, corresponding to the exact mass of the organic motif. The positive mode ESI-MS of **1** and **2** revealed molecular ion peaks (*m*/*z*) at 417.06 and 494.15, respectively. [Ni(HL1)(OAc)(H_2_O)]^+^, 417.06 (fit: 417.0), indicated one HL1 ligand and the OAc^−^ might be a discrete species obtained from the neutral molecule under electrospray ionization conditions. Meanwhile, [NiL2(CH_3_OH)_3_(H_2_O)]^+^, 494.9 (fit: 494.6), manifested that one L2^−^ anion was presumably ionized from the parent species under electrospray ionization conditions.

### 2.2. Crystal Structure

**Figure 1:**
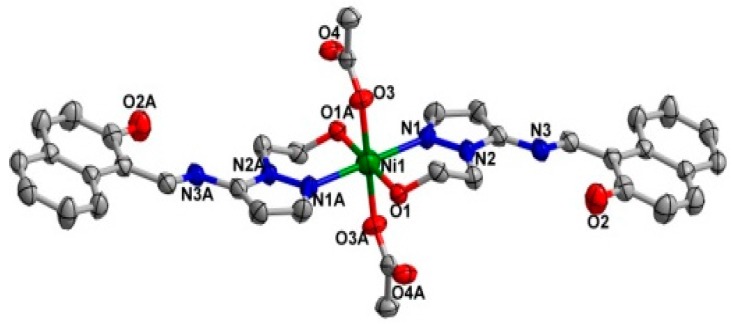
X-ray crystal structure of complex **1** along with atomic numbering, hydrogen atoms have been omitted for clarity.

**Figure 2:**
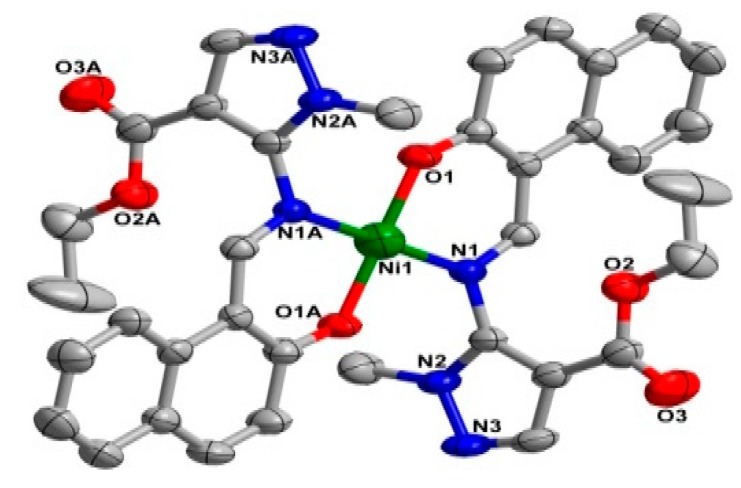
X-ray crystal structure of complex **2** along with atomic numbering, hydrogen atoms have been omitted for clarity.

The two coordination complexes had a mononuclear structure. Compound **1** is a monoclinic species with space group *P*2_1_/*n*. As shown in Figure 1, the Ni(II) atom is six-coordinated in an octahedral coordination geometry, with the equatorial plane consisting of four O atoms, O1, O1A from two different ligands, and O3, O3A from two carboxylate anions, while the axial positions are occupied by two nitrogen atoms (N1, N1A) from two ligands. The bond lengths of Ni1-N1 for the equatorial plane are longer than those of Ni1-O3 for the axial positions. The system of **2** is monoclinic and the space group is *P*2_1_/c; Ni(II) centre was coordinated by two *trans*-chelating HL2 anions with two nitrogen atoms (N1, N1A) and two oxygen atoms (O1, O1A) (Figure 2). Furthermore, the N1, O1, N1A, O1A of the two ligands and Ni centre are situated in the equatorial plane. The selected bond angles of **1** and **2** were all close to 90°. For **1**, the Ni–O distances ranging from 2.052 (2) to 2.0632 (2) Å are shorter than Ni–N distance (2.094 (2) Å). Meanwhile, the Ni–O distance in **2** is 1.822 (2) Å, which is shorter than Ni–N distance (1.891 (2) Å). The results indicate strong interactions between oxygen and Ni. The crystal data as well as details of data collection and refinement details have been given in Table 1, while selected bond lengths and bond angles for **1** and **2** are listed in Table 2 and Table 3, respectively.

### 2.3. DNA Binding Studies

#### 2.3.1. Electronic Absorption Spectral Studies

**Figure 3:**
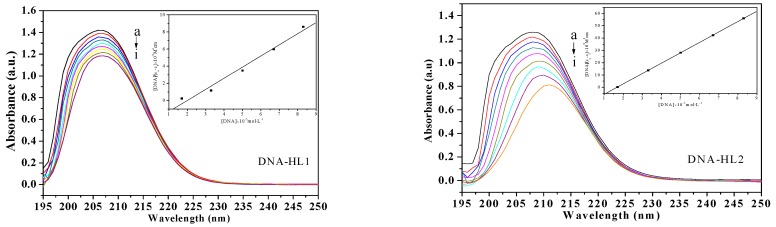

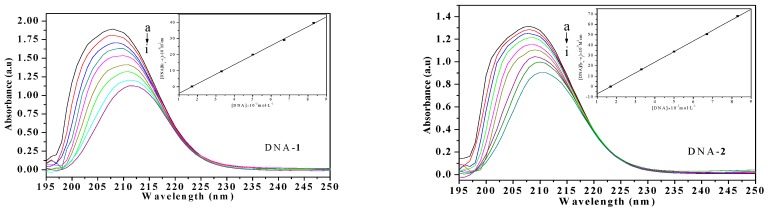
Absorption spectra of HL1, HL2, **1** and **2** (1.0 × 10^−7^ mol·L^−1^) in the absence and presence of increasing amounts of CT-DNA (from a to i) at room temperature. The arrow shows the absorption changes on increasing CT-DNA concentration. Insert: Plot of [DNA]/(*ε*_a_ − *ε*_f_) versus [DNA] for absorption titration of CT-DNA with the compound.

The UV-Vis spectra of the compounds (HL1, HL2, **1**, and **2**) and calf thymus DNA (CT-DNA) were measured to examine the binding mode. As shown in Figure 3, the absorption peak at about 210 nm is attributed to intraligand π → π* transitions. As the concentration of CT-DNA was increased, various hypochromisms were observed. Meanwhile, the bathochromic shift trends were remarkable except for ligand HL1. The extent of hypochromism commonly parallels the strength of intercalative interaction [22]. The red-shifts are due to the insertion into DNA base pairs and the hypochromism is observed [23,24].

To elucidate and compare the binding strength of the four compounds with CT-DNA quantitatively, the intrinsic binding constant *K*_b_ were calculated according to the Benesi-Hildebrand equation [25], as follows:

(1) $$\left[\mathrm{DNA}\right]/\left(\varepsilon_{a}-\varepsilon_{f}\right)=\left[\mathrm{DNA}\right]/\left(\varepsilon_{b}-\varepsilon_{f}\right)+1/K_{b}(\varepsilon_{b}-\varepsilon_{f})$$

where [DNA] is the concentration of DNA in base pairs, *ε*_a_, *ε*_f_ and *ε*_b_ are the observed extinction coefficient of (*A*_obsd_/[complex]), the free compound and the absorbance of the compound in the fully DNA-bound form, respectively. The ratio of slope to intercept of a plot (inset in Figure 3) of [DNA]/(*ε*_a_ − *ε*_f_) versus [DNA] gives the value of *K*_b_ of ligands HL1, HL2, **1** and **2** (**1** > **2** > HL2 > HL1), which were estimated to be 4.30 × 10^3^ M^−1^ (*R* = 0.954), 5.89 × 10^3^ M^−1^ (*R* = 0.998), 5.95 × 10^3^ M^−1^ (*R* = 0.999), and 5.92 × 10^3^ M^−1^ (*R* = 0.997), respectively, the order of magnitude is similar to the document reported [19]. The *K*_b_ value of the two Ni(II) complexes are higher than their free ligands. The results indicate that the Ni(II) complexes can intercalate into CT-DNA strongly, which may be related to their coordinate structural characteristics. Moreover, the exact binding mode cannot be merely confirmed by electronic absorption spectral studies.

#### 2.3.2. Circular Dichroic Spectral Studies

Circular Dichroic (CD) spectral titration analysis was also applied to assess whether DNA molecules undergo conformational changes induced by the complexes. The CD spectra of CT-DNA showed one positive band at 276 nm due to base stacking and one negative band at 246 nm due to the helicity of B-type DNA [26]. With the addition of HL1 and HL2 to the solution of CT-DNA, the intensity of both the positive and negative peaks decreased slightly (shown in Figure S2). When the two Ni complexes were added, the intensity of all negative peaks at 246 nm obviously decreased. In addition, the positive peak at 276 nm increased greatly with the addition of **1**, indicating **1** has a certain degree of effect on the stacking of DNA base pairs and double helix structure and **2** has more effect on double helix structure. Perhaps the B-DNA conformation is transformed to the Z-DNA conformation [27]. However, the B-DNA is still the main, because absorption peaks in other locations are not detected [28].

#### 2.3.3. Fluorescence Spectroscopic Studies

The addition of HL1, HL2, **1** and **2** into the GelRed/CT-DNA binary binding system induced a significant fluorescence quenching. The peak emission was at around 600 nm when excited by 350 nm wavelength light which indicated that GelRed molecules intercalated between the contiguous base pairs of CT-DNA and were sufficiently protected from the fluorescence quenching by polar solvent molecules. The emission spectra of the GelRed/CT-DNA system in the absence and presence of the compounds (0–9.0 × 10^−5^ mol·L^−1^) were shown in Figure S3. While adding each compound to DNA pretreated with GelRed, the DNA-induced emission intensity around 600 nm was decreased. These results strongly suggested the existence of competitive intercalative binding between GelRed and **1**, **2** and their ligands HL1, HL2 in which they could displace the GelRed molecules from the DNA neighboring base pairs after push the GelRed molecules back into the aqueous solution and the intercalative binding mode of the test compounds to DNA was similar to that of GelRed [29]. Moreover, the quenching ability of all complexes to GelRed fluorescence can be quantitatively assessed by their respective quenching constant, *K*_SV_, which was derived from the Stern-Volmer quenching equation [30]:

(2) $$I_{0}/I=1+K_{SV}[Q]$$

where *I*_0_ is the emission intensity in the absence of complex, *I* is the emission intensity in the presence of compound, *K*_SV_ is the quenching constant, and [*Q*] is the concentration of the compound. The *K*_SV_ values for ligands HL1, HL2, **1** and **2** were 1.19 × 10^3^ L·mol^−1^, 3.82 × 10^3^ L·mol^−1^, 5.73 × 10^3^ L·mol^−1^, 5.57 × 10^3^ L·mol^−1^ (**1** > **2** > HL2 > HL1), respectively. Both **1** and **2** exhibited more intensive intercalation with DNA than their corresponding ligands, which is probably attributable to the potential synergistic effect when their ligands are coordinated to the nickel metal ion.

### 2.4. Bovine Serum Albumin Binding Studies

#### 2.4.1. UV Absorption Spectra of Bovine Serum Albumin

To analyse the method of quenching of bovine serum albumin (BSA) with HL1, HL2, **1** and **2**, the absorption spectra of BSA in the absence and presence of the compound were recorded at room temperature, as shown in Figure S4. There are two absorption bands in the UV spectrum of the solution of BSA, which are respectively referred to the content of *α*-helix [31] and the π → π* transition of the aromatic amino acids (Trp, Tyr, and Phe) [32]. With the addition of the compound to BSA, the absorption peak at 203 nm is obviously decreased with different degree, whereas the intensity of the peak at 278 nm is slightly increased. These results suggested that the interaction of all compounds with BSA may cause conformational changes in BSA.

#### 2.4.2. Circular Dichroism Spectra of BSA

The CD spectra of BSA exhibited two negative bands in the UV region at 208 and 222 nm which are contributed by the π → π* and *n* → π* transition of peptide bonds in the α-helix [33]. The CD spectra of BSA in the absence and presence of increasing amount of HL1, HL2, **1** and **2** were recorded, as shown in Figure S5. The results have been revealed in terms of mean residue ellipticity (MRE) in deg·cm^2^·dmol^−1^ by [34]:

(3) $$\mathrm{MRE}=\theta_{obs}\;(m\;\deg)/(10\times n\times l\times C_{p})$$

where *θ*_obs_ is the CD in millidegree, *n* is the number of amino acid residue (583 for BSA), *l* is the path length (1 cm), *C*_P_ is the molar concentration of the albumin. Figure S5 shows that the binding of all compounds to BSA leads to a decrease of band intensity without any significant shift of the peak. The *α*-helical contents of free and combined BSA were also calculated from MRE values at 208 nm using equation [35]:

(4) $$a\%\;\mathrm{helix}=[(-{\mathrm{MRE}}_{208}-4000)/(33,000-4000)]\times 100$$

where 4000 is the MRE value of β-form and random coil conformation and 33,000 is the MRE value of a pure α-helix at 208 nm. MRE_208_ is the observed MRE value at 208 nm. A decreasing α-helices content tendency was estimated with the increasing concentration of compounds (Table 4), implying the loss of biological activity of the protein which is associated with its secondary structure contents. It is worth noting that the effect of **2** is prominent.

#### 2.4.3. Fluorescence Quenching of Bovine Serum Albumin

**Figure 4:**
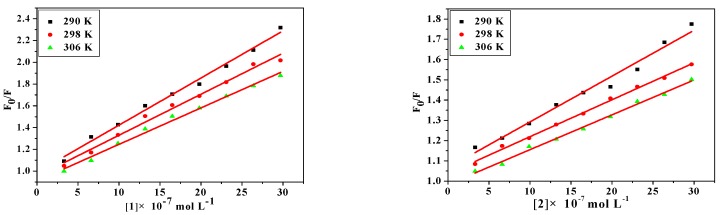

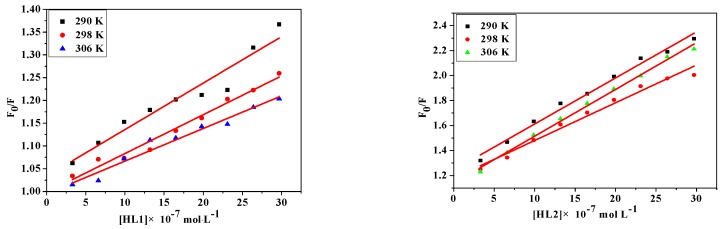
Stern-Volmer plots of the fluorescence quenching of BSA by HL1, HL2, **1** and **2** at different temperatures: 290 K, 298 K, 306 K.

In order to get more insight into the binding affinity of all compounds with BSA, emission titrations are an effective method to qualitatively analyze the protein folding mechanism and association reactions [36]. BSA has the three fluorophores, namely, tryptophan, tyrosine and phenylalanine, inserted in the first subdomain IB and subdomain IIA, respectively. When excited at 280 nm, BSA exhibits a strong fluorescence emission with a peak at 346 nm due to the tryptophan residues, because phenylalanine has a rather low yield of quantum and tyrosine is almost quenched when it is ionized or close to a tryptophan residue, an amino group, or a carbonyl group [37]. On increasing the concentration of complexes, the fluorescence emission intensities of BSA at 346 nm were regularly decreased when excited at 280 nm, meanwhile, no shift and other changes are observed (Figure S6) [38]. To further explore the mechanism of the static quenching, the decrease in intensity is described by the Stern-Volmer equation [39]:

(5) $$\frac{F_{0}}{F}=1+K_{SV}[Q]=1+k_{q}\tau_{0}[Q]$$

where [*Q*] is the concentration of quencher, *F*_0_ and *F* are the fluorescence intensities in the absence and presence of quencher, *K*_SV_ is the Sterne-Volmer quenching constant, *k*_q_ is the biomolecular quenching constant and *τ*_0_ is the average lifetime of protein in the absence of quencher (*τ*_0_ = 10^−8^ s). From the quenching plot of *F*_0_/*F* vs. [*Q*] (Figure 4), *K*_SV_ and *k*_q_ of the compounds obtained from the slope in 290 K, 298 K and 306 K are listed in Table 5. The values of *K*_sv_ are gradually decreased with the increase of the temperature, which is possibly because the increasing temperature contributes to accelerating the thermal motion of molecules to reduce the stability and associated complex decomposition [40]. Meanwhile, all values of *k*_q_ are larger than the maximum dynamic quenching constant (2.00 × 10^12^ M^−1^·s^−1^) [41], indicating that the probable quenching mechanism is static quenching.

**Figure 5:**
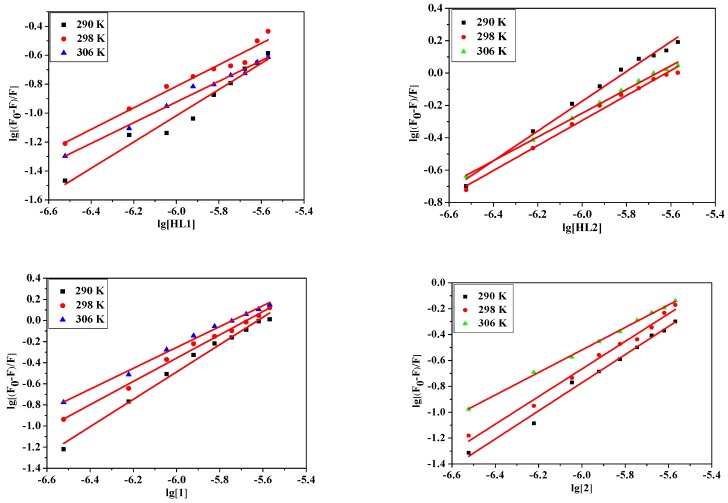
The logarithmic plots of the fluorescence quenching of BSA by HL1, HL2, **1** and **2** at different temperatures: 290 K, 298 K, 306 K.

For the static quenching interaction, the apparent binding constant (*K*) and the number of binding sites (*n*) for the complex can also be determined according to the Scatchard equation:

(6) $$\mathrm{lg}(F_{0}-F)/F=\mathrm{lg}K+n\mathrm{lg}[Q]$$

where *F*_0_ and *F* represent the fluorescence intensities in the absence and presence of quencher, respectively. *K* is the BSA quencher binding constant, *n* is the number of binding sites. The *n* and *K* can be calculated by the slope and the intercept of the double logarithm regression curve of log[(*F*_0_ − *F*)/*F*] vs. log [*Q*] (Figure 5). The calculated values of *K* and *n* are listed in Table 6. It is observed that the values of *K* decrease sharply with the increase of the temperature, which firmly confirms that the quenching of compounds and BSA is static. The numbers of binding sites (*n*) of all complexes are about equal to 1, suggesting the high affinity binding sites of albumins. This result confirms the effect of substitution on binding with BSA is 1:1 at a certain of concentration. Compound **1** has a higher magnitude of binding than other complexes.

### 2.5. Determination of Thermodynamic Parameters

The thermodynamic parameters for the binding between drug molecule and biological macromolecules such as BSA can provide valuable information to predict the mode of interaction between them. Some possible interaction forces in this regard are: coulombic, hydrophobic and van der Waals interaction [42]. The thermodynamic parameters viz., standard enthalpy change (*ΔH*^0^) and standard entropy change (*ΔS*^0^) (Table 6) were calculated from Van’t Hoff equation [43]:

(7) $$lnK=-\Delta H^{0}/RT+\Delta S^{0}/R$$

(8) $$\Delta G^{0}=\Delta H^{0}-T\Delta S^{0}$$

where *R* is the gas constant and *K* is the binding constant at corresponding temperature, and *ΔG*^0^ is the free energy change which is estimated from above equation. The negative values of *ΔG*^0^ at constant pressure and temperature indicates that the processes of interaction are spontaneous. A negative value of *ΔH*^0^ reveals that the binding process is exothermic in nature. The *ΔH*^0^ < 0 and *ΔS*^0^ < 0 values suggest that the interaction forces between complexes and BSA are mainly van der Waals interactions or hydrogen bonds.

### 2.6. In Vitro Studies

#### 2.6.1. Methyl Thiazolyl Tetrazolium (MTT) Assay

The in vitro cytotoxicity of HL1, HL2, **1** and **2** were evaluated by MTT assays on eight cell lines (HepG2, MGC80-3, T-24, BEL-7404, NCI-H460, SK-OV-3, A549, and HL-7702). Under the same experimental conditions, cisplatin was used as a positive control. The inhibition rates and *IC*_50_ values of cisplatin and all complexes against the eight cell lines are listed in Table 7 and Table 8, respectively. All displayed *IC*_50_ values in the micromolar range. Specifically, the inhibition ratio of **1** (40 μM) was almost the highest in all cell lines all the time (up to 80–90%) and about to the same that of cisplatin, but the choice of specificity is not certain. Meanwhile, **2** also displayed significantly enhanced cytotoxicity against the tested cell lines. The cytotoxicity of all complexes was further quantified by determining their corresponding *IC*_50_ values. The *IC*_50_ values for **1** and **2** are statistically lower than that for metal-free ligands HL1 and HL2 in all of the tested cells, suggesting that coordinated Ni(II) atoms play a notable role in biological properties of the complexes. What’s more, **1** had nearly equivalent *IC*_50_ values when compared with values for cisplatin against the same cell lines. It is worthwhile to mention that **1** shows higher cytotoxicity towards A549 cells than cisplatin. When we compare the *IC*_50_ values, it can be found that the *IC*_50_ values of **1** are lower than those of **2** for all the cancer cell lines, indicating that **1** possessed better activities than **2** under identical experimental conditions.

#### 2.6.2. Cell Cycle Arrest

**Figure 6:**
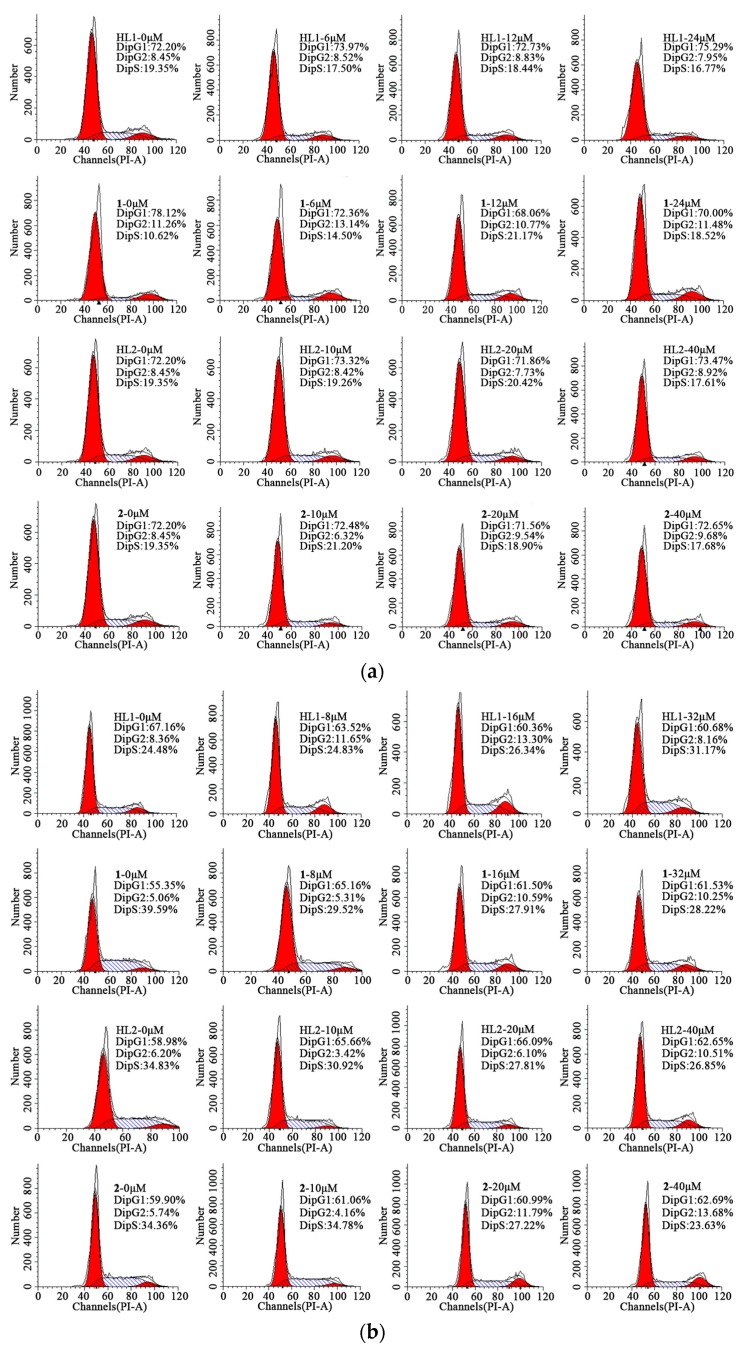
Cell cycle arrest. (**a**) Effects of HL1, HL2, **1**, and **2** on the cell cycle of HepG2 cells after incubation for 48 h; (**b**) Effects of HL1, HL2, **1**, and **2** on the cell cycle of MGC80-3 cells after incubation for 48 h.

To determine the intracellular mechanism of the growth inhibition of tumor cells by HL1, HL2, **1** and **2**, a cell cycle assay was investigated by flow cytometric analysis of PI-stained cells. As shown in Figure 6, the HepG 2 and MGC80-3 tumor cells were incubated with all complexes of gradient concentrations (0, 6, 12, 24 μM) for 48 h. This significant increase in the apoptotic tumor cells strongly suggested that **1** induced efficient apoptosis in HepG 2 cells perturbation of the cell cycle and trapped the cells via the induction of DNA damage in the S phase. Unluckily, no significant changes in the proportion of the cells in the whole phases were observed in the HepG 2 cells with HL1, HL2 and **2**. In addition, this observed increases occurred at the expense of a decrease in the proportion of the MGC80-3 tumor cells in the S, G1, G1/G2 and G2 phase respectively when treated with HL1, HL2, **1** and **2**.

#### 2.6.3. Cell Apoptosis

**Figure 7:**
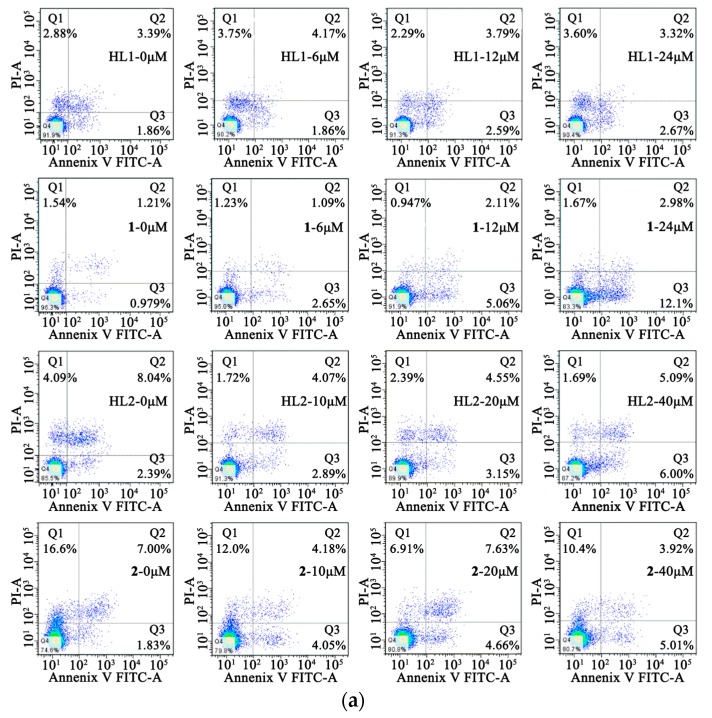

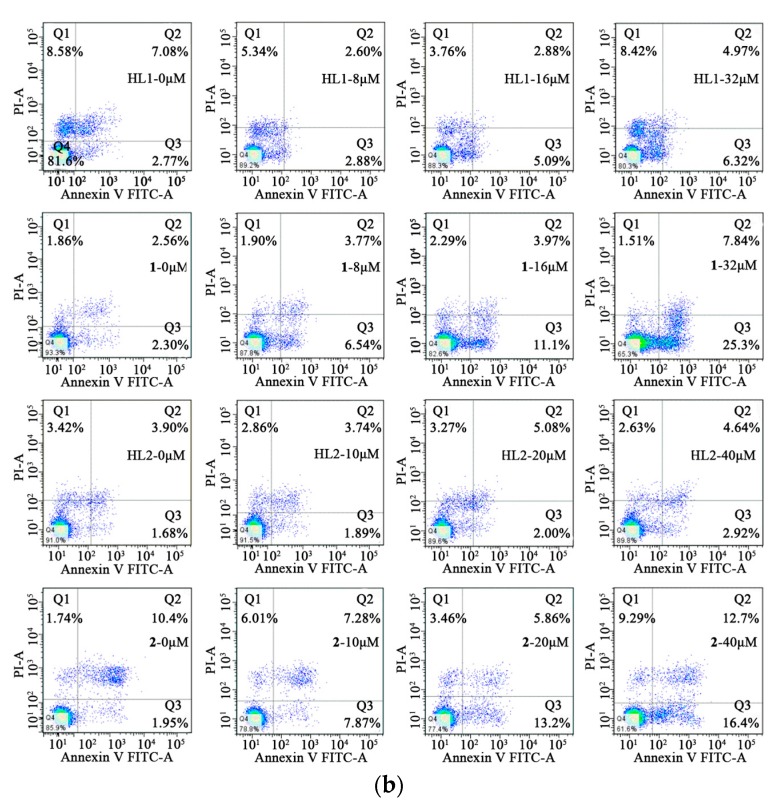
Cell apoptosis. (**a**) Effects of HL1, HL2, **1** and **2** on the cell early apoptosis of HepG2 cells after incubation for 24 h; (**b**) Effects of HL1, HL2, **1** and **2** on the cell early apoptosis of MGC80-3 cells after incubation for 24 h.

In order to determine the inhibition of cell growth caused by the induction of apoptosis, Annexin V-FITC/PI staining was used. The HepG 2 cells were treated with HL1 and **1** at different concentrations (0, 6, 12, 24 μM) for 24 h while MGC80-3 tumor cells were treated with the concentrations (0, 8, 16, 32 μM) for 24 h. At the same time, the HepG 2 and MGC80-3 tumor cells were incubated with **2** and ligand HL2 at different concentrations (0, 10, 20, 40 μM) for 24 h. This resulted in an increase in apoptotic cells including early apoptotic cells and late apoptotic cells. As shown in Figure 7, **1** induced a higher incidence of early apoptosis. 

The percentage of early apoptosis of HepG 2 was 0.979%, 2.65%, 5.06%, 12.1%, respectively. And the percentage of early apoptosis of HepG 2 was positive correlation of the concentration of 1, but it is not notable for HL1, HL2 and **2**. As is seen, the viability of MGC80-3 tumor cells decreases in HL1, HL2, **1**, and **2** as a function of concentration indicating a dose-dependent growth inhibitory effect, particularly in the two Ni(II) complexes. The percentage of early apoptosis of MGC80-3 treated with **1** and **2** were 2.30%, 6.54%, 11.1%, 25.3% and 1.95%, 7.87%, 13.2%, 16.4%, respectively. In a word, complexation with Ni(II) significantly improves the anticancer effect of the parent ligand.

## 3. Materials and Methods

### 3.1. General Information

All reagents and solvents were all of analytical grade and were utilized without any further purification. Calf thymus DNA (CT-DNA), bovine serum albumin (BSA), and GelRed were obtained from commercially available sources and used as received. Doubly distilled water was used in the whole process. All complexes including ligands and complexes were dissolved in DMSO as the stock solution (2 × 10^−3^ mol·L^−1^), and diluted properly as the working solution.

Elemental analysis was performed by a Series II CHNS/O Analyzer 2400 II (PerkinElmer, Waltham, MA, USA). The IR spectra were recorded on a Spectrum One FT-IR spectrophotometer (PerkinElmer, Waltham, MA, USA) with KBr pressed disks. UV-Vis spectra and electronic spectra in solution were obtained in a 1.0 cm quartz cuvette on a Cary 60 UV-Visible spectrophotometer (Agilent, Santa Clara, CA, USA). UV-Vis spectra in solid state were recorded on a UV-2600 instrument (Shimadzu, Tokyo, Japan). The fluorescence study was determined by a RF-5310PC spectrofluorophotometer (Shimadzu, Tokyo, Japan). The CD spectra were acquired with a Jasco-810 spectropolarimeter (Jasco, Tokyo, Japan). The crystals for single-crystal X-ray analyses and property measurements were prepared by the following methods. Electrospray ionization mass spectrometry (ESI-MS) was measured using an LCQ/AD Quadrupole Ion Trap mass spectrometer (ThermoFinngan, Waltham, MA, USA).

### 3.2. Chemistry

#### 3.2.1. Synthesis of the Ligands HL1 and HL2

**Scheme 1:**
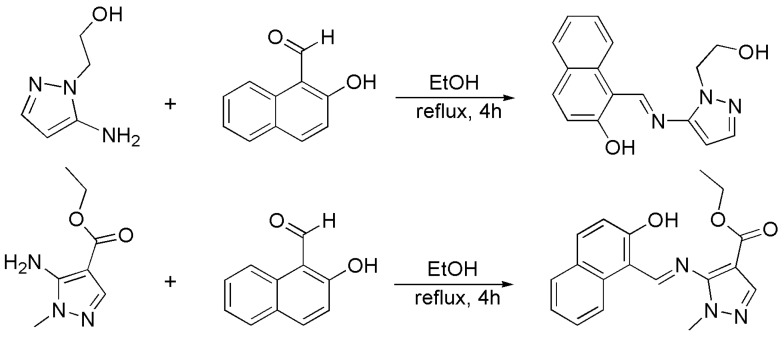
Synthesis of the ligands HL1 and HL2.

The two Schiff base ligands were prepared based on the literature [44,45]. As shown in Scheme 1, a mixture of 5-amino-1-(2-hydroxyethyl)pyrazole (2 mmol, 254 mg), 2-hydroxy-1-naphthaldehyde (2 mmol, 350 mg) and 5 drops of the glacial acetic acid in the ethanol solution (30 mL) was vigorously stirred and refluxed at 80 °C for 4 h. 

The solvent was evaporated, yielding yellow needle-shaped crystals the product HL1 (Scheme 1). HL2 was synthesized by the same method, but replacing the 5-amino-1-(2-hydroxyethyl)pyrazole with 5-amino-1-methylpyrazolyl-4-carboxylic acid methyl ester.

HL1: yellow needle-shaped crystals. Yield 80%. Anal. Calcd. (%) for C_16_H_15_N_3_O_2_ (281.31): C, 68.25; H, 5.33; N, 14.93. Found (%): C, 68.32; H, 5.26; N, 14.89. ESI-MS *m*/*z*: [HL1-H]^−^, 280.0. FT-IR (KBr, cm^−1^): 3203br, 1622vs, 1602vs, 1572vs, 1078vs, 819vs, 767vs, 1486s, 1465s, 1335s, 1422m, 1360m, 1525m, 1295m, 1179m, 1126m, 969w, 573w, 531w.

HL2: yellow schistose crystals. Yield 76%. Anal. Calcd. (%) for C_18_H_17_N_3_O_3_ (323.35): C, 66.80; H, 5.26; N, 12.99. Found (%): C, 66.86; H, 5.20; N, 12.86. ESI-MS *m*/*z*: [HL2-H]^−^, 321.9. FT-IR (KBr, cm^−1^): 3430br, 1691vs, 1624vs, 1529vs, 1248vs, 1143vs, 1601s, 1571s, 1483m, 1259m,1182m, 1056m, 1423w, 1404w, 859w, 718w.

#### 3.2.2. Synthesis of Ni(HL1)_2_(OAc)_2_ (**1**)

Complex **1** was synthesized by adding nickel acetate tetrahydrate (0.5 mmol, 0.125 g) into a 15 mL absolute ethanol solution of an equimolar amount of the corresponding ligand HL1 and refluxing at 80 °C for 4 h. After the resulting solution was filtered and left to evaporate slowly at room temperature after several days to afford yellow block crystals suitable for X-ray diffraction analysis in 75% yield (based on Ni(II)). Anal. Calcd. (%) for C_36_H_36_NiN_6_O_8_ (739.42): C, 58.42; H, 4.87; N, 11.36. Found (%): C, 58.28; H, 4.68; N, 11.83. FT-IR (KBr phase, cm^−1^): 3420br, 1573vs, 1416vs,1621s, 1602s, 1485s, 1328s, 1128m, 816m, 748m, 666m, 983w, 967w, 911w, 713w.

#### 3.2.3. Synthesis of [Ni(L2)_2_](**2**)

In the reaction, nickel nitrate hexahydrate (0.3 mmol, 0.088 g), HL2 (0.3 mmol, 0.097 g) and ethanol (15 mL) were put into a 23 mL Teflon-lined stainless steel autoclave followed by three drops of triethylamine solution. The mixture was sealed, heated up to 80 °C and kept for 72 h. Then the autoclave was allowed to cool down to room temperature at a rate of 5 °C. The dark green block crystals of X-ray quality were washed with ethanol. Yield: 70% (based on Ni(II)). Anal. Calcd. (%) for C_36_H_32_NiN_6_O_6_ (703.39): C, 61.42; H, 4.55; N, 11.94. Found (%): C, 61.48; H, 4.25; N, 12.02. FT-IR (KBr phase, cm^−1^): 3421br, 2984vs, 1064vs, 1693s, 1618m, 1603m, 1537m, 1232m,896w, 836w, 780w, 601w.

### 3.3. Crystal Structure Determination and Refinement

X-ray single-crystal diffraction data for **1** and **2** (The Cambridge Structural Database (CCDC) #1525665 and #1525666) were collected with a Bruker SMART CCD instrument (Bruker, Baden-Württemberg, Germany) using graphite monochromatic Mo *Kα* (λ = 0.71073 Å) at 293(2) K. Structures were solved by direct methods by using the program SHELXS-97 and refined by SHELXL-97 [46,47]. All non-hydrogen atoms were refined anisotropically by weighted full- matrix least-squares on *F*^2^ using the SHELXTL-97 program [48]. H atoms on C atoms were included in the difference Fourier map and were refined isotropically with a riding model. DIAMOND (version 3.2.11.0, Crystal Impact GbR, Bonn, Germany) [49] was applied for molecular graphics.

### 3.4. Stability Studies

All the complexes (2 × 10^−3^ mol·L^−1^, 30 μL) prepared were added into 2970 μL Tris-HCl-NaCl buffer (5 mM Tris-HCl and 50 mM NaCl, pH 7.35 at 25 °C) and the mixtures were kept for 0 h, 12 h, 24 h, 36 h, and 48 h at room temperature, respectively.

### 3.5. DNA Binding Experiments

In order to preferably understand the mechanism of drug action between DNA and metallodrugs, four experiments as follows were carried out.

#### 3.5.1. UV-Vis Spectra

Electronic absorption titration experiment was performed to explore the DNA binding with the ligands and complexes. The experiments including CT-DNA were conducted in Tris buffer followed by diluted suitably to get the appropriate concentrations and kept at 277 K for shorter than a week. The CT-DNA stock solution with a ratio of UV absorbance at 260 and 280 nm (A_260_/A_280_ = 1.89), indicating that the CT-DNA was sufficiently free of protein [50]. The concentration of DNA was determined via the UV absorbance at 260 nm, taking *ε*_260_ as 6600 M^−1^·cm^−1^ [51]. The absorption titrations were done at a constant concentration of all complexes, while gradually increasing the concentration of CT-DNA. Equal volume solution of CT-DNA was respectively added to the prepared solution and the reference solution to eliminate the DNA absorbance. After each addition, the complex and CT-DNA mixtures were allowed to incubate at ambient temperature for 5 min. The intrinsic binding constant *K*_b_ is also monitored from the spectroscopic titration data.

#### 3.5.2. Circular Dichroism

The CD spectra of CT-DNA in the buffer solution at 6.7 × 10^−5^ mol·L^−1^ in the absence and presence of 1.7 × 10^−7^ mol·L^−1^ HL1, HL2, **1** and **2** were recorded in Tris buffer at 25.0 ± 0.1 °C and scanned in the range of 220–320 nm. The solutions were incubated for 5 min after each addition of equivalent complexes solutions. The spectrum only containing Tris buffer served as the blank sample. Then, the generated CD spectra were subtracted from the blank sample spectra for data analysis.

#### 3.5.3. Competitive Binding Experiments by Fluorescence Spectral Analysis

The binding abilities of all complexes to CT-DNA were further studied by a competing assay using GelRed (GR) as an intercalative binding probe. GR, environmentally safe and ultra-sensitive, is a newly developed DNA intercalator which can replace the classic DNA intercalator ethidium bromide (EB). Furthermore, both GelRed and EB bound with CT-DNA show characteristic fluorescence at about 590 nm when excited at 350 nm [52]. The stock solutions of all complexes were prepared with the concentration of 1 mM. In competitive binding experiments, the solutions of GelRed and CT-DNA mixture ([GR]/[DNA] = 1:10) were pre-incubated at 277 K overnight to ensure sufficient interactions and revealed peak emission at around 590 nm when excited by 350 nm wavelength light. The variation of emission spectra of the GelRed-CT-DNA system was recorded by the addition of a 30 μL of the complexes solutions step by step into the 3.0 mL solution of GelRed and CT-DNA mixture. The fluorescence quenching curves of GelRed bound to CT-DNA by HL1, HL2, **1** and **2** (0–9.0 × 10^−5^ mol·L^−1^) at room temperature were carried out.

### 3.6. Bovine Serum Albumin Binding Studies

#### 3.6.1. UV-Vis Spectra

The BSA stock solution was prepared in 50 mM phosphate buffer saline (PBS)/100 mM NaCl buffer at pH 7.4 to attain a final concentration of 1 μM, and stored at 4 °C with constant ionic strength. The PBS buffer (3 mL) as a reference solution was used in the experiment. A BSA solution (3 mL, 1 × 10^−6^ M) was titrated by successive additions of the stock solutions of each complex (1 × 10^−3^ M) and the variations in the BSA absorption were recorded after each addition. The UV-Vis absorption titration spectrum was scanned on the range of wavelength from 195 to 315 nm.

#### 3.6.2. Circular Dichroism

For the CD experiments, the concentrations and parameters of BSA and each complex were the same like in the above experiment. The CD spectra of BSA in the absence and presence of all complexes were recorded in the range of 200~250 nm at 298 K using a bandwidth of 1 nm. The CD spectrum of PBS buffer was subtracted from the sample spectra for data analysis.

#### 3.6.3. Fluorescence Spectra

In the fluorescence quenching experiment, the concentrations and parameters of BSA and each complex were the same as the above experiments and the fluorescence intensity was recorded from 290 to 490 nm at 290, 298 and 306 K. An excitation and corresponding emission was at 280 nm and 348 nm, respectively. The excitation slit was set at 5 nm and emission slit was set at 2.5 nm throughout the study. When excited at 296 nm, the fluorescence spectra of BSA exhibited a maximum emission at 346 nm corresponded to tryptophan residues (Trp-212). Quenching of the tryptophan residues of BSA at 343 nm was monitored by keeping the concentration of BSA constant and using the increasing concentration of complexes [53,54], in order to make the solutions with the multiple mole ratio of the complexes to BSA.

### 3.7. MTT Assay

The anticancer activities of HL1, HL2, **1** and **2** against seven cancer cell lines (HepG2, MGC80-3, T-24, BEL-7404, NCI-H460, SK-OV-3, and A549), and the normal liver cell line HL-7702 were measured by the MTT assay, using cisplatin as a reference metallodrug. All complexes were dissolved in DMSO to prepare 2 mM concentration the stock complex solutions. The cancer cells were seeded at a density of 4500 cells/well in the collagen-coated 96 well plate and grown with PPMI 1640 medium overnight at 37 °C in a 5% CO_2_ incubator. All cells were treated with 40 μM concentrations for 48 h and cell viability was evaluated by MTT assay. Then 10 μL of 5 mg/mL MTT solution was added to each well, and cells were incubated for an additional 4 h. 100 μL of anhydrous DMSO was added to dissolve the formed formazan crystals and shaken carefully for 5 min. The absorbance was read with an enzyme labeling instrument with 490 nm. Then the percentage of cell growth inhibition was calculated by the following formula:

% inhibition=[mean OD of untreated cells (control)mean OD of treated cells (control)]×100.

The cytotoxicity was evaluated according to *IC*_50_ values which were calculated using the Bliss method (*n* = 5). All the tests were repeated by at least three independent experiments.

### 3.8. Cell Cycle Arrest 

HepG2 and MGC80-3 cells were incubated with different concentrations of HL1 and **1** (0, 6, 12, 24 μM for 48 h) while HL2 and **2** (0, 10, 20, 40 μM for 48 h). Then cells were harvested and washed with cold PBS twice, and fixed with ice-cold 70% ethanol at −20 °C overnight. For staining, fixed cells were resuspended in 1 mL of PBS containing PI (50 μg/mL, 0.5 mL) and Rnase A (10 μg/mL, 25 μL) at 37 °C in the dark for 30 min. The cell cycle distribution was analyzed by a FACS Aria II flow cytometer (BD, New York, NY, USA).

### 3.9. Apoptosis Study by Flow Cytometry

Cell apoptosis was detected by Annexin V-FITC/PI staining with flow cytometric analysis [55]. Briefly, HepG2 and MGC80-3 cells were collected after treatment which was performed as the cell cycle experiment. Then the cells were washed with PBS, (100 μL 1 × binding buffer, 5 μL Annexin V-FITC and 5 μL PI) and incubated in the dark at room temperature for 20 min. Finally, 400 μL 1 × binding buffer was added, and the stained cells were tested by flow cytometry.

## 4. Conclusions

Two new mononuclear Ni(II) complexes have been synthesized and characterised by spectroscopic studies to explore the influence of pyrazole Shiff-base complexes on the structure, and reactivity towards the bio-molecules BSA and CT-DNA as well as in vitro cytotoxicity activities. The Ni(II) complexes’ structures were established by X-ray crystallographic studies. The reactions of HL1, HL2, **1** and **2** with BSA/CT-DNA and cytotoxicity were determined by various methods. The quenching with BSA was found to be static. The present study highlighted that the coordination with Ni(II) can increase DNA/BSA-binding ability and the anticancer activity in vitro. Among the four compounds, complex **1** exhibited better binding affinity with BSA and DNA. Meanwhile, the cytotoxic studies showed that **1** displays promising cytotoxic activity against eight cell lines (HepG2, MGC80-3, T-24, BEL-7404, NCI-H460, SK-OV-3, A549, and HL-7702), which are almost similar to those of cisplatin. Our results suggested that **1** may be a potential metallodrugs candidate for treatment of some certain diseases. We find that in an aqueous medium **1** is more likely to be an ionic species through loss of an acetate moiety when compared to HL1, HL2 and **2**. The features of **1** may lead to the better binding and cytotoxic properties. The findings should be valuable to study the necessity of the coordination action for the biological properties and show that other complexes of metals other than Pt also have the potential to serve as effective anticancer drugs.

## Figures and Tables

**Table 1:** Crystallographic data and structure refinement parameters for **1** and **2**.

Complex	1	2
Empirical formula	C_36_H_36_NiN_6_O_8_	C_36_H_32_NiN_6_O_6_
Formula weight (*M*)	739.42	703.39
Temperature (K)	293 (2)	293 (2)
Crystal system	Monoclinic	Monoclinic
Space group	*P*2_1_/*n*	*P*2_1_/*c*
*a* (Å)	14.0548 (5)	12.6852 (8)
*b* (Å)	7.9911 (2)	11.0140 (6)
*c* (Å)	15.2876 (6)	11.8301 (8)
*α* (°)	90.00	90.00
*β* (°)	105.902 (4)	100.008 (6)
*γ* (°)	90.00	90.00
Volume (Å^3^)	1651.30 (10)	1627.69 (17)
Z	2	2
Calculated density (Mg/m^3^)	1.487	1.435
Absorption coefficient (mm^−1^)	0.65	0.65
*F*(000)	772.0	732.0
*θ* range for data collection	3.8 to 24	3.7 to 24.7
Limiting indices	−17 ≤ *h* ≤ 17	−15 ≤ *h* ≤ 15
	−9 ≤ *k* ≤ 9	−13 ≤ *k* ≤ 13
	−19 ≤ *l* ≤ 18	−14 ≤ *l* ≤ 14
Reflections collected	8684	16088
Independent reflections	3374 [*R*_int_ = 0.0436]	3325 [*R*_int_ = 0.0907]
Data/restraints/parameters	3374/2/237	3325/0/225
Goodness-of-fit on *F*^2^	1.031	1.062
Final *R* indices [*I* > 2*σ*(*I*)]	*R*_1_ = 0.0478	*R*_1_ = 0.0528
	*ωR*_2_ = 0.0957	*ωR*_2_ = 0.1206
*R* indices (all data)	*R*_1_ = 0.0804	*R*_1_ = 0.0832
	*ωR*_2_ = 0.1119	*ωR*_2_ = 0.1429

**Table 2:** Selected bond lengths (Å) and angles (°) for **1**.

Ni1–O1	2.052 (2)	O3–Ni1–N1A	90.12 (9)
Ni1–O3	2.0632 (2)	O3A–Ni1–N1A	89.88 (9)
Ni1–N1	2.094 (2)	O1A–Ni1–N1	89.83 (9)
O1A–Ni1–O3A	89.43 (8)	O1A–Ni1–N1A	90.17 (9)
O1–Ni1–O3A	90.57 (8)		

Symmetry transformations used to generate equivalent atoms: (A) −x + 2, −y + 1, −z + 1.

**Table 3:** Selected bond lengths (Å) and angles (°) for **2**.

Ni1–O1	1.822 (2)	O1–Ni1–N1	92.13 (10)
Ni1–N1	1.891 (2)	O1–Ni1–N1A	87.87 (10)

Symmetry transformations used to generate equivalent atoms: (A) −x + 2, −y + 2, −z.

**Table 4:** The content of α-helix of bovine serum albumin (BSA) in the absence of and presence of HL1, HL2, **1**, and **2**.

System (%)	[ML]:[BSA] 0:1	[ML]:[BSA] 0.5:1	[ML]:[BSA] 1:1
BSA-HL1	51.16	48.57	46.88
BSA-1	51.16	41.67	38.14
BSA-HL2	51.16	44.38	42.23
BSA-2	51.16	44.49	40.15

**Table 5:** The Stern-Volmer quenching constants of BSA by HL1, HL2, **1** and **2** at different temperature: 290 K, 298 K, 306 K.

System	T (K)	*K*_SV_ (×10^5^ L·mol^−1^)	*k*_q_ (×10^13^ L·mol^−1^·S^−1^)	*R* ^a^	SD ^b^
BSA-HL1	290	3.36	3.36	0.9319	0.00320
	298	2.81	2.81	0.9771	0.00152
	306	2.36	2.36	0.9621	0.00165
BSA-**1**	290	14.31	14.31	0.9908	0.00488
	298	12.44	12.44	0.9999	0.00078
	306	11.06	11.06	0.9915	0.00362
BSA-HL2	290	11.93	11.93	0.9275	0.01174
	298	9.96	9.96	0.9791	0.00514
	306	4.12	4.12	0.9998	0.00079
BSA-**2**	290	7.47	7.47	0.9782	0.00394
	298	6.02	6.02	0.9962	0.00132
	306	5.68	5.68	0.9997	0.00080

*R* ^a^ is correlation coefficient for the *K*_SV_ values. SD ^b^ is the standard deviation for the *K*_SV_ values.

**Table 6:** The binding constants and thermodynamic parameters of BSA by HL1, HL2, **1** and **2** at different temperatures: 290 K, 298 K, 306 K.

System	T (K)	*K* (L·mol^−1^)	*n*	Δ*G*^0^ (kJ·mol^−1^)	Δ*H*^0^ (kJ·mol^−1^)	Δ*S*^0^ (J·mol^−1^·K^−1^)
BSA-HL1	290	2.65 × 104	0.91	−24.04	−112.30	−304.34
	298	4.43 × 103	0.74	−21.61		
	306	2.33 × 103	0.71	−19.17		
BSA-**1**	290	1.93 × 107	1.30	−40.09	−187.51	−508.35
	298	1.83 × 106	1.10	−36.02		
	306	3.33 × 105	0.99	−31.95		
BSA-HL2	290	1.77 × 105	0.92	−28.42	−116.21	−302.71
	298	2.22 × 104	0.77	−26.00		
	306	1.43 × 104	0.73	−23.58		
BSA-**2**	290	5.60 × 105	1.09	−32.87	−122.24	−308.16
	298	5.35 × 105	1.07	−30.41		
	306	3.97 × 104	0.87	−27.94		

**Table 7:** Growth inhibition of HL1, HL2, **1** and **2** against different cell lines in vitro.

	HepG 2	MGC80-3	T-24	BEL-7404	NCI-H460	SK-OV-3	A549	HL-7702
HL1	62.82 ± 1.46	41.57 ± 0.19	53.83 ± 0.33	36.00 ± 5.91	28.69 ± 0.18	50.24 ± 2.65	19.74 ± 0.05	61.25 ± 0.47
HL2	62.23 ± 0.93	39.11 ± 0.80	53.43 ± 0.09	27.04 ± 0.87	34.98 ± 0.05	55.62 ± 0.07	27.63 ± 1.12	58.75 ± 2.51
**1**	94.01 ± 1.22	90.07 ± 0.03	92.82 ± 0.60	74.53 ± 0.17	85.13 ± 0.42	89.34 ± 1.75	76.37 ± 0.48	92.48 ± 1.06
**2**	73.45 ± 0.28	59.30 ± 0.31	54.63 ± 0.49	45.31 ± 0.55	31.45 ± 0.41	53.76 ± 3.20	28.65 ± 5.15	65.44 ± 0.64
Cisplatin	90.59 ± 0.41	88.28 ± 0.33	80.40 ± 0.63	73.99 ± 0.20	72.45 ± 0.13	68.19 ± 4.43	52.80 ± 0.55	91.18 ± 0.17

**Table 8:** *IC*_50_ values of HL1, HL2, **1** and **2** on different cell lines in vitro.

	HepG2	MGC80-3	T-24	BEL-7404	NCI-H460	SK-OV-3	A549	HL-7702
HL1	31.17 ± 2.06	93.85 ± 1.96	36.77 ± 0.09	>100	>100	45.36 ± 1.17	>100	29.15 ± 0.14
HL2	30.59 ± 1.01	88.25 ± 3.12	38.85 ± 0.14	>100	>100	39.80 ± 0.74	>100	26.77 ± 1.58
**1**	13.33 ± 0.11	15.73 ± 0.02	18.18 ± 0.05	24.07 ± 0.12	24.84 ± 0.08	16.16 ± 0.39	13.26 ± 0.20	14.95 ± 0.05
**2**	23.18 ± 0.01	37.84 ± 1.53	28.84 ± 0.42	46.59 ± 0.79	>100	36.80 ± 4.62	>100	23.75 ± 0.91
Cisplatin	3.93 ± 0.01	14.61 ± 0.06	17.22 ± 0.13	20.41 ± 0.05	14.84 ± 0.11	10.09 ± 1.11	35.29 ± 0.42	7.86 ± 0.10

## Supplementary Materials

Supplementary Materials are available online.

## References

[1] Barceló-Oliver M., García-Raso Á., Terrón A., Molins E., Prieto M.J., Moreno V., Martínez J., Lladó V., López’ I., Gutiérrez A. (2007). Synthesis and mass spectroscopy kinetics of a novel ternary copper(II) complex with cytotoxic activity against cancer cells. J. Inorg. Biochem..

[2] Rosenberg B., Van Camp L., Krigas T. (1965). Inhibition of cell division in *Escherichia coli* by electrolysis products from a platinum electrode. Nature.

[3] Kelland L. (2007). The resurgence of platinum-based cancer chemotherapy. Nat. Rev. Cancer.

[4] Suntharalingam K., Johnstone T.C., Bruno P.M., Lin W., Hemann M.T., Lippard S.J. (2013). Bidentate ligands on osmium(VI) nitrido complexes control intracellular targeting and cell death pathways. J. Am. Chem. Soc..

[5] Santini C., Pellei M., Gandin V., Porchia M., Tisato F., Marzano C. (2014). Advances in copper complexes as anticancer agents. Chem. Rev..

[6] Kate A.N., Kumbhar A.A., Khan A.A., Joshi P.V., Puranik V.G. (2013). Monitoring cellular uptake and cytotoxicity of copper(II) complex using a fluorescent anthracene thiosemicarbazone ligand. Bioconj. Chem..

[7] Gaynor D., Griffith D.M. (2012). The prevalence of metal-based drugs as therapeutic or diagnostic agents: Beyond platinum. Dalton Trans..

[8] Chang H., Jia L., Xu J., Zhu T., Xu Z., Chen R., Ma T., Wang Y., Wu W. (2016). Syntheses, crystal structures, anticancer activities of three reduce Schiff base ligand based transition metal complexes. J. Mol. Struct..

[9] Inam A., Siddiqui S.M., Macedo T.S., Moreira D.R.M., Leite A.C.L., Soares M.B.P., Azam A. (2014). Design, synthesis and biological evaluation of 3-[4-(7-chloro-quinolin-4-yl)-piperazin-1-yl]-propionic acid hydrazones as antiprotozoal agents. Eur. J. Med. Chem..

[10] Júnior W.B., Alexandre-Moreira M.S., Alves M.A., Perez-Rebolledo A., Parrilha G.L., Castellano E.E., Piro O.E., Barreiro E.J., Lima L.M., Beraldo H. (2011). Analgesic and anti-inflammatory activities of salicylaldehyde 2-chlorobenzoyl hydrazone (H_2_LASSBio-466), salicylaldehyde 4-chlorobenzoyl hydrazone (H_2_LASSBio-1064) and their zinc(II) complexes. Molecules.

[11] Muregi F.W., Ishih A. (2010). Next-generation antimalarial drugs: Hybrid molecules as a new strategy in drug design. Drug Dev. Res..

[12] Mohamed G.G., Zayed E.M., Hindy A.M. (2015). Coordination behavior of new bis Schiff base ligand derived from 2-furan carboxaldehyde and propane-1,3-diamine. Spectroscopic, thermal, anticancer and antibacterial activity studies. Spectrochim. Acta Part A.

[13] Pfaltz A., Lancaster J.R. (1988). The Bioinorganic Chemistry of Nickel.

[14] Alexiou M., Tsivikas I., Dendrinou-Samara C., Pantazaki A.A., Trikalitis P., Lalioti N., Kyriakidis D.A., Kessissoglou D.P. (2003). High nuclearity nickel compounds with three, four or five metal atoms showing antibacterial activity. J. Inorg. Biochem..

[15] Kurtaran R., Yıldırım L.T., Azaz A.D., Namli H., Atakol O. (2005). Synthesis, characterization, crystal structure and biological activity of a novel heterotetranuclear complex: [NiLPb (SCN)_2_(DMF)(H_2_O)]_2_, bis-{[μ-*N*,*N*′-bis(salicylidene)-1,3-propanediaminato-aqua-nickel(II)](thiocyanato)(μ-thiocyanato)(μ-*N*,*N*′-dimethylformamide) lead(II)}. J. Inorg. Biochem..

[16] Luo W., Meng X., Sun X., Xiao F., Shen J., Zhou Y., Cheng G., Ji Z. (2007). Synthesis, crystal structure and bioactivity of a novel linear trinuclear nickel(II) complex. Inorg. Chem. Commun..

[17] Afrasiabi Z., Sinn E., Lin W., Ma Y., Campana C., Padhye S. (2005). Nickel(II) complexes of naphthaquinone thiosemicarbazone and semicarbazone: Synthesis, structure, spectroscopy, and biological activity. J. Inorg. Biochem..

[18] Buschini A., Pinelli S., Pellacani C., Giordani F., Ferrari M.B., Bisceglie F., Giannetto M., Pelosi G., Tarasconi P. (2009). Synthesis, characterization and deepening in the comprehension of the biological action mechanisms of a new nickel complex with antiproliferative activity. J. Inorg. Biochem..

[19] Kalaivani P., Saranya S., Poornima P., Prabhakaran R., Dallemer F., Vijaya Padma V., Natarajan K. (2014). Biological evaluation of new nickel(II) metallates: Synthesis, DNA/protein binding and mitochondrial mediated apoptosis in human lung cancer cells (A549) via ROS hypergeneration and depletion of cellular antioxidant pool. Eur. J. Med. Chem..

[20] Raj P., Singh A., Singh A., Singh N. (2017). Syntheses and Photophysical properties of Schiff-Base Ni(II) Complexes: Application for Sustainable Antibacterial activity and Cytotoxicity. ACS Sustain. Chem. Eng..

[21] Prabhakaran R., Jayabalakrishnan C., Krishnan V., Pasumpon K., Sukanya D., Bertagnolli H., Natarajan K. (2006). Preparation, spectral characterization, electrochemistry, EXAFS, antibacterial and catalytic activity of new ruthenium(III) complexes containing ONS donor ligands with triphenylphosphine/arsine. Appl. Organomet. Chem..

[22] Krishnamoorthy P., Sathyadevi P., Butorac R.R., Cowley A.H., Bhuvanesh N.S., Dharmaraj N. (2012). Copper(I) and nickel(II) complexes with 1:1 vs. 1:2 coordination of ferrocenyl hydrazone ligands: Do the geometry and composition of complexes affect DNA binding/cleavage, protein binding, antioxidant and cytotoxic activities?. Dalton Trans..

[23] Tysoe S.A., Morgan R.J., Baker A.D., Strekas T.C. (1993). Spectroscopic investigation of differential binding modes of Δ- and Λ-Ru (bpy)_2_(ppz)^2+^ with calf thymus DNA. J. Phys. Chem..

[24] Rajarajeswari C., Ganeshpandian M., Palaniandavar M., Riyasdeen A., Akbarsha M.A. (2014). Mixed ligand copper(II) complexes of 1,10-phenanthroline with tridentate phenolate/pyridyl/(benz) imidazolyl Schiff base ligands: Covalent vs. non-covalent DNA binding, DNA cleavage and cytotoxicity. J. Inorg. Biochem..

[25] Niu M., Hong M., Cheng S., Dou J. (2014). Effect of structure and composition of nickel(II) complexes with salicylidene Schiff base ligands on their DNA/protein interaction and cytotoxicity. J. Inorg. Biochem..

[26] Schäfer S., Ott I., Gust R., Sheldrick W.S. (2007). Influence of the Polypyridyl (pp) Ligand Size on the DNA Binding Properties, Cytotoxicity and Cellular Uptake of Organoruthenium(II) Complexes of the Type [(η^6^-C_6_Me_6_)Ru(L)(pp)]^n+^ [L = Cl, *n* = 1; L = (NH_2_)_2_CS, *n* = 2]. Eur. J. Inorg. Chem..

[27] Richards A.D., Rodger A. (2007). Synthetic metallomolecules as agents for the control of DNA structure. Chem. Soc. Rev..

[28] Chen Z., Wang X., Zhu Y., Li Y., Guo Z. (2007). Selective guanosine binding and cytotoxicity of a benzimidazole derived dinickel complex. J. Inorg. Biochem..

[29] Zhang G., Guo J., Zhao N., Wang J. (2010). Study of interaction between kaempferol—Eu^3+^ complex and DNA with the use of the Neutral Red dye as a fluorescence probe. Sens. Actuators B.

[30] Qiu B., Guo L., Chen Z., Chi Y., Zhang L., Chen G. (2009). Synthesis of *N*-4-butylamine acridone and its use as fluorescent probe for ctDNA. Biosens. Bioelectron..

[31] Ruiz J., Vicente C., de Haro C., Bautista D. (2013). Novel bis-*C*,*N*-cyclometalated iridium(III) thiosemicarbazide antitumor complexes: Interactions with human serum albumin and DNA, and Inhibition of cathepsin B. Inorg. Chem..

[32] Haghighi F.H., Hadadzadeh H., Darabi F., Jannesari Z., Ebrahimi M., Khayamian T., Salimi M., Rudbari H.A. (2013). Polypyridyl Ni(II) complex, [Ni(tppz)_2_]^2+^: Structure, DNA- and BSA binding and molecular modeling. Polyhedron.

[33] Zhao L., Liu R., Zhao X., Yang B., Gao C., Hao X., Wu Y. (2009). New strategy for the evaluation of CdTe quantum dot toxicity targeted to bovine serum albumin. Sci. Total Environ..

[34] Wang Q., Liu P., Zhou X., Zhang X., Fang T., Liu P., Min X., Li X. (2012). Thermodynamic and conformational investigation of the influence of CdTe QDs size on the toxic interaction with BSA. J. Photochem. Photobiol. A.

[35] Xiao Q., Huang S., Qi Z., Zhou B., He Z., Liu Y. (2008). Conformation, thermodynamics and stoichiometry of HSA adsorbed to colloidal CdSe/ZnS quantum dots. Biochim. Biophys. Acta.

[36] Pan B.F., Gao F., Ao L.M. (2005). Investigation of interactions between dendrimer-coated magnetite nanoparticles and bovine serum albumin. J. Magn. Magn. Mater..

[37] Sułkowska A. (2002). Interaction of drugs with bovine and human serum albumin. J. Mol. Struct..

[38] Jana S.K., Seth S.K., Puschmann H., Hossain M., Dalai S. (2014). Synthesis and X-ray structure of a new zinc(II) coordination polymer: Interaction with DNA and double stranded RNA and elucidation of the molecular aspects of the binding to bovine serum albumin. RSC Adv..

[39] Rajendiran V., Karthik R., Palaniandavar M., Stoeckli-Evans H., Periasamy V.S., Akbarsha M.A., Srinag B.S., Krishnamurthy H. (2007). Mixed-ligand copper(II)-phenolate complexes: Effect of coligand on enhanced DNA and protein binding, DNA cleavage, and anticancer activity. Inorg. Chem..

[40] Gelamo E.L., Tabak M. (2000). Spectroscopic studies on the interaction of bovine (BSA) and human (HSA) serum albumins with ionic surfactants. Spectrochim. Acta Part A.

[41] Ware W.R. (1962). Oxygen quenching of fluorescence in solution: An experimental study of the diffusion process. J. Phys. Chem..

[42] Leckband D. (2000). Measuring the forces that control protein interactions. Annu. Rev. Biophys. Biomol. Struct..

[43] Tong J., Tian F., Li Q., Li L., Xiang C., Liu Y., DAI J., Jiang F. (2012). Probing the adverse temperature dependence in the static fluorescence quenching of BSA induced by a novel anticancer hydrazine. Photochem. Photobiol. Sci..

[44] Benkö Z., Burck S., Gudat D., Nieger M., Nyulászi L., Shore N. (2008). Pyrido-annellated diazaphospholenes and phospholenium ions. Dalton Trans..

[45] Upadhyay A., Vaidya S., Venkatasai V.S., Jayapal P., Srivastava A.K., Shanmugam M., Shanmugam M. (2013). Synthesis and characterization of 3d and 4f metal complexes of Schiff base ligands. Polyhedron.

[46] Sheldrick G.M. (1990). Phase annealing in SHELX-90: Direct methods for larger structures. Acta Crystallogr. Sect. A Found. Crystallogr..

[47] Sheldrick G.M. The SHELX Homepage. http://shelx.uni-ac.gwdg.de/SHELX/.

[48] Sheldrick G.M. (2008). A short history of SHELX. Acta Crystallogr. Sect. A Found. Crystallogr..

[49] Brandenburg K., Putz H. DIAMOND, Program for Crystal and Molecular Structure Visualization. http://www.crystalimpact.com/diamond/.

[50] Chand D.K., Schneider H.J., Bencini A., Bianchi A., Giorgi C., Ciattini S., Valtancoli B. (2015). Affinity and nuclease activity of macrocyclic polyamines and their Cu(II) complexes. Chem. Eur. J..

[51] Reichmann M.E., Rice S.A., Thomas C.A., Doty P. (1954). A further examination of the molecular weight and size of desoxypentose nucleic acid. J. Am. Chem. Soc..

[52] Saleh M., Soliman H., Elmatbouli M. (2008). Loop-mediated isothermal amplification as an emerging technology for detection of *Yersinia ruckeri* the causative agent of enteric red mouth disease in fish. BMC Vet. Res..

[53] Joseph R.L., Lakowicz R. (1999). Principles of Fluorescence Spectroscopy.

[54] Hu Y.J., Liu Y., Wang J.B., Xiao X.H., Qu S.S. (2004). Study of the interaction between monoammonium glycyrrhizinate and bovine serum albumin. J. Pharm. Biomed. Anal..

[55] Chou C.C., Yang J.S., Lu H.F., Ip S.W., Lo C., Wu C.C., Lin J.P., Tang N.Y., Chung J.G., Chou M.J., Teng Y.H., Chen D.R. (2010). Quercetin-mediated cell cycle arrest and apoptosis involving activation of a caspase cascade through the mitochondrial pathway in human breast cancer MCF-7 cells. Arch. Pharm. Res..

