# Supplementary material for: Ni(II) Complexes with Schiff Base Ligands: Preparation, Characterization, DNA/Protein Interaction and Cytotoxicity Studies
Source: Molecules. 2017 Oct 24;22(10):1772. doi: 10.3390/molecules22101772 (PMC6151616; doi:10.3390/molecules22101772)
Supplement: Supplementary file 1 [file molecules-22-01772-s001.pdf]

Supporting Information for

Ni(II) Complexes with Schiff Base Ligands: Preparation, Characterization, DNA/Protein Interaction and Cytotoxicity Studies

Hui Yu [1,2], Wei Zhang [2], Qing Yu [2], Fu-Ping Huang [2,*], He-Dong Bian [1,2,*] and Hong Liang [2]

[1] School of Chemistry and Chemical Engineering, Guangxi Key Laboratory of Chemistry and Engineering of Forest Products, Guangxi University for Nationalities, Nanning 530008, China; yh9930228@163.com

[2] School of Chemistry and Pharmacy, Key Laboratory for the Chemistry and Molecular Engineering of Medicinal Resources (Ministry of Education of China), Guangxi Normal University, Guilin 541004, China; ZW574873607@163.com (W.Z.); gxqbs_qing@163.com (Q.Y.); hliang@gxnu.edu.cn (H.L.)

* Correspondence: huangfp2010@163.com (F.-P.H.); gxunchem@163.com (H.-D.B.)

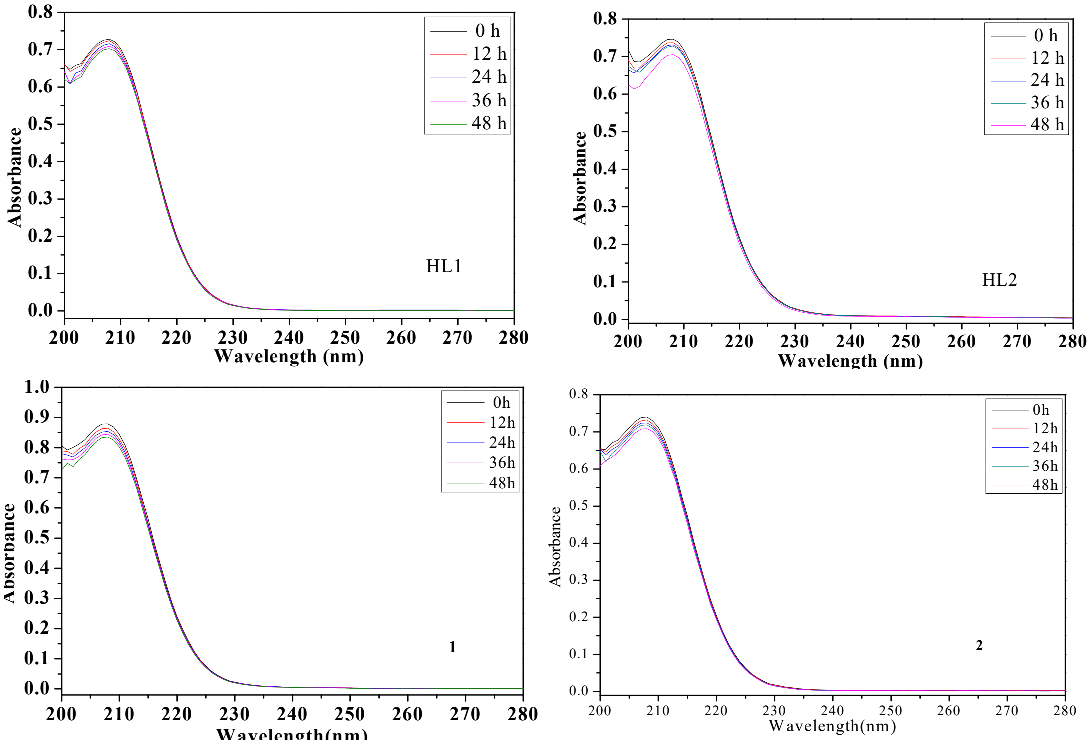

Figure S1. UV/Vis absorbance of HL1, HL2, **1**, and **2** in solution after standing in different times at ambient temperature.

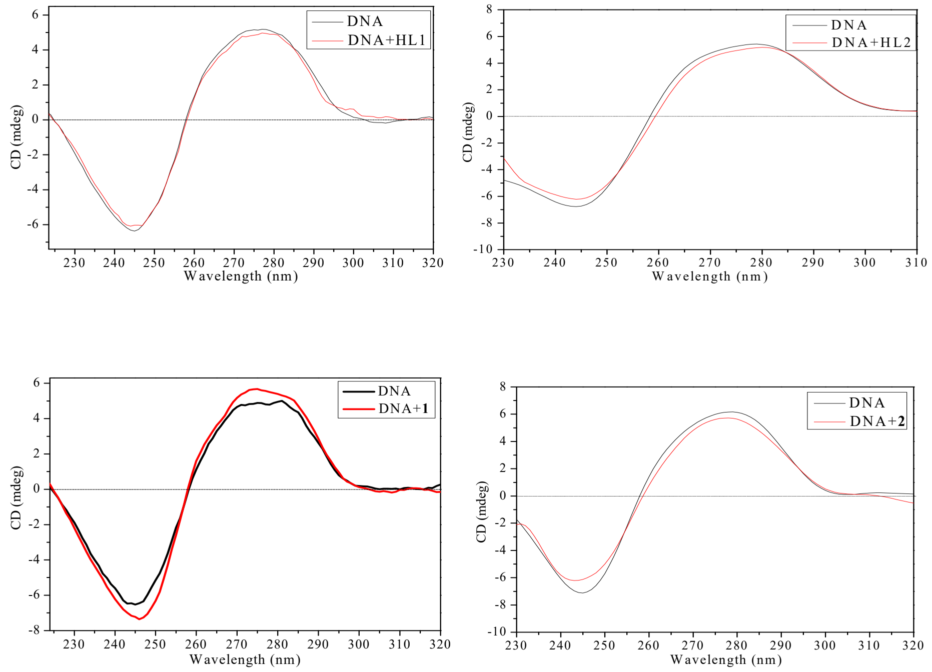

Figure S2. The CD spectra of CT-DNA in the buffer solution at 6.7×10^{-5} mol·L^{-1} in the absence and presence of 1.7×10^{-7} mol·L^{-1} HL1, HL2, **1**, and **2**.

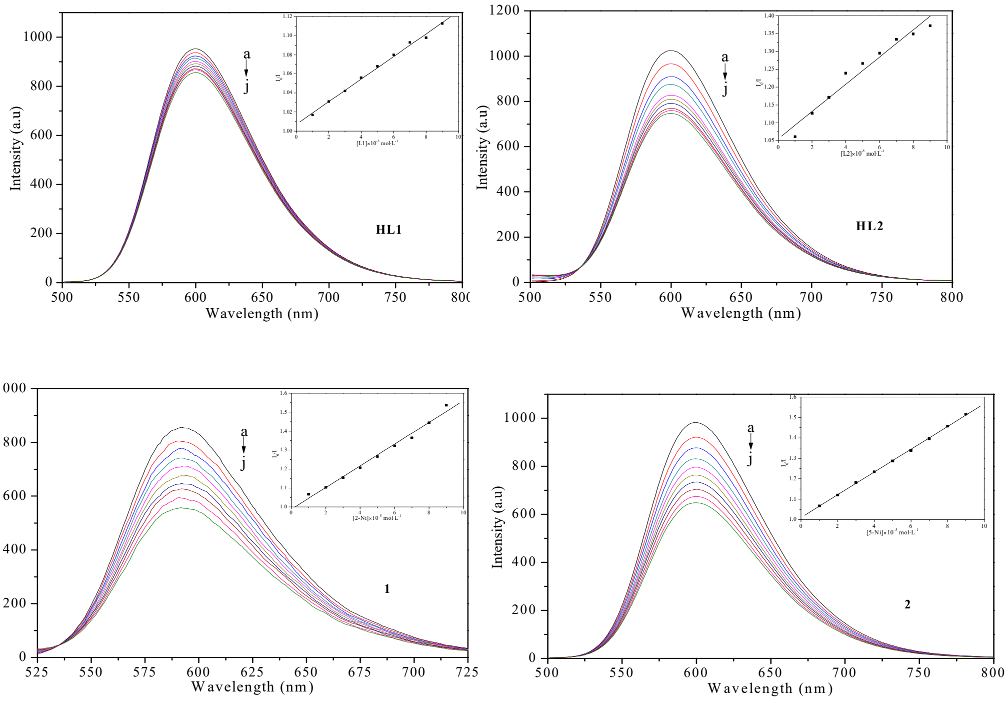

Figure S3. The Fluorescence quenching curves of GelRed bound to CT-DNA by HL1, HL2, **1**, and **2** (0-9.0 × 10^{-5} mol·L^{-1}) at room temperature. Insert: Plot of I_0/I versus [compound].

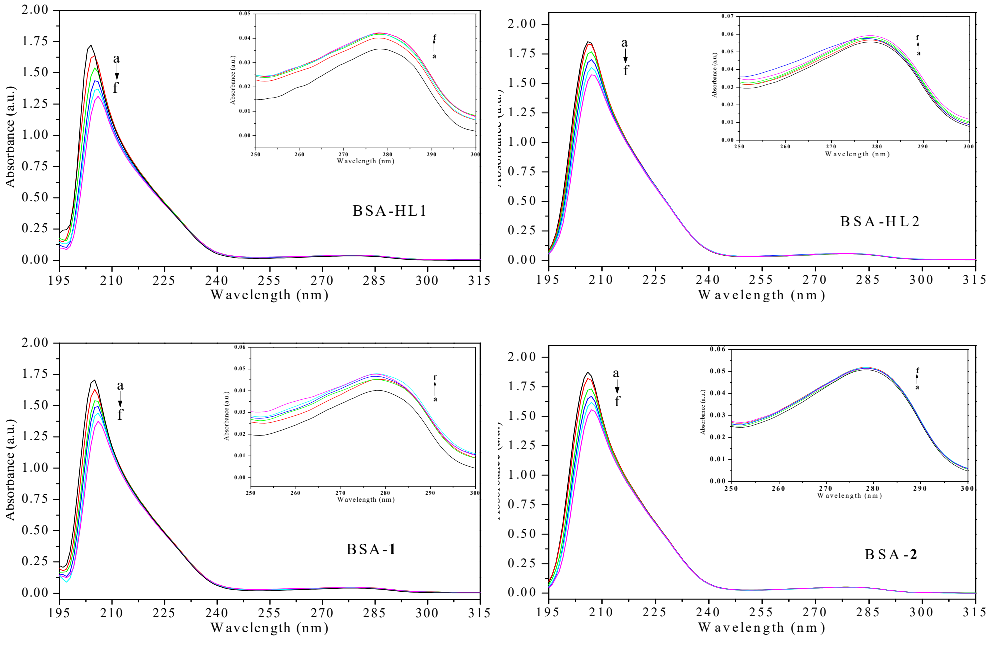

Figure S4. Absorption spectra of BSA (1.0 × 10^{-6} mol·L^{-1}) in the absence and presence of increasing amount of HL1, HL2, **1**, and **2**. The concentration of HL1, HL2, **1**, and **2** were varied from 0 to 1.0 × 10^{-8},2.0 × 10^{-8}, 3.0 × 10^{-8}, 4.0 × 10^{-8} and 5.0 × 10^{-8} mol·L^{-1} respectively, as a step of 1 ×10^{-8} mol·L^{-1}.

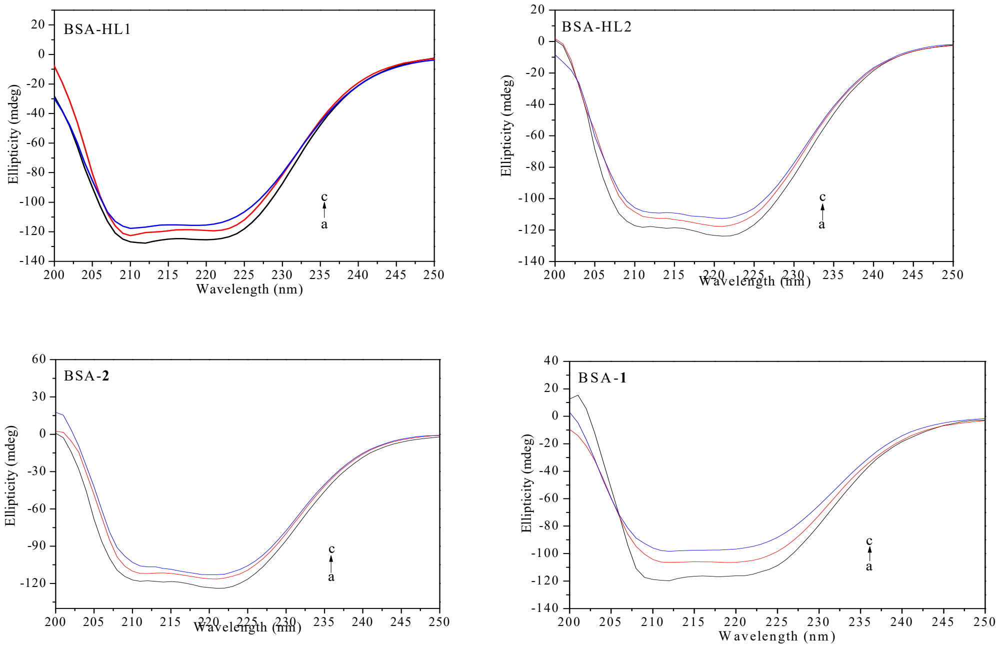

Figure S5. The CD spectra of BSA (1.0×10^{-6} mol·L^{-1}) in the absence and presence of increasing amount of HL1, HL2, **1**, and **2**. From a to c, the ratios of [BSA] : [complex] were 1: 0, 1: 0.5 and 1:1.

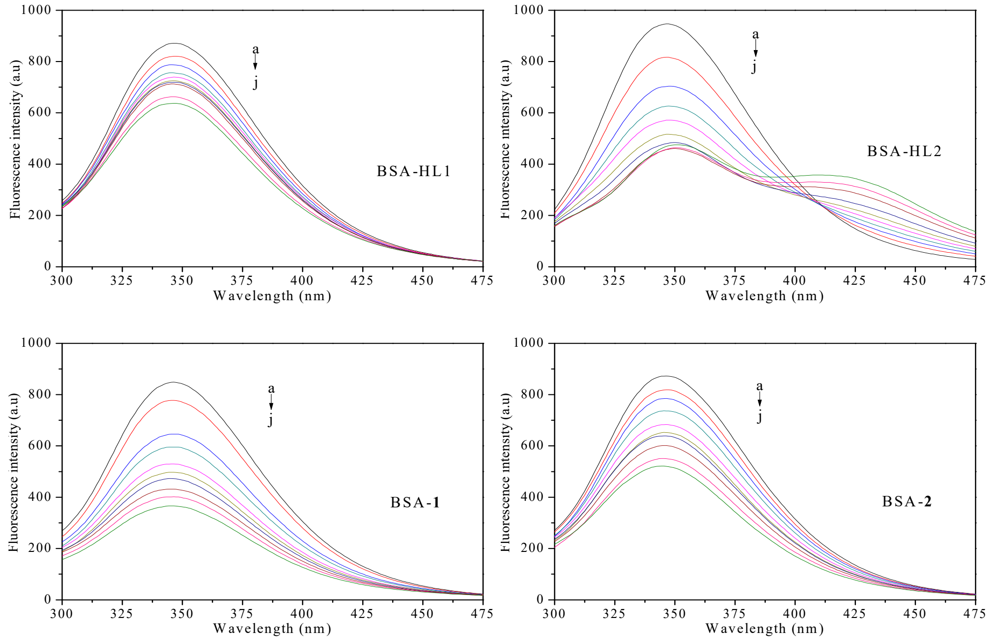

Figure S6. Emission spectra of BSA in the absence and the presence of HL1, HL2, **1**, and **2**at various concentrations, T = 290 K and BSA concentration was 1.0×10^{-6} mol·L^{-1}. From a to j, the concentrations of HL1, HL2, **1**, and **2** were varied from 0 to 3.0×10^{-6} mol·L^{-1}, as a step of 3.0×10^{-7} mol·L^{-1}.
